# A case of premortem diagnosis of cardiac tamponade due to pericardial metastasis of rectal cancer

**DOI:** 10.1093/omcr/omad039

**Published:** 2023-04-20

**Authors:** Yuki Takano, Junichi Mazaki, Koichiro Tasaki, Ryutaro Udo, Tomoya Tago, Naoto Okazaki, Kenta Kasahara, Hiroshi Kuwabara, Masanobu Enomoto, Tetsuo Isizaki, Jun Matsubayashi, Toshitaka Nagao, Yuichi Nagakawa, Kenji Katsumata, Akihiko Tsuchida

**Affiliations:** Department of Gastrointestinal and Pediatric Surgery, Tokyo Medical University, Shinjuku, Tokyo 1600023, Japan; Department of Gastrointestinal and Pediatric Surgery, Tokyo Medical University, Shinjuku, Tokyo 1600023, Japan; Department of Anatomic Pathology, Tokyo Medical University, Shinjuku, Tokyo 1600023, Japan; Department of Gastrointestinal and Pediatric Surgery, Tokyo Medical University, Shinjuku, Tokyo 1600023, Japan; Department of Gastrointestinal and Pediatric Surgery, Tokyo Medical University, Shinjuku, Tokyo 1600023, Japan; Department of Gastrointestinal and Pediatric Surgery, Tokyo Medical University, Shinjuku, Tokyo 1600023, Japan; Department of Gastrointestinal and Pediatric Surgery, Tokyo Medical University, Shinjuku, Tokyo 1600023, Japan; Department of Gastrointestinal and Pediatric Surgery, Tokyo Medical University, Shinjuku, Tokyo 1600023, Japan; Department of Gastrointestinal and Pediatric Surgery, Tokyo Medical University, Shinjuku, Tokyo 1600023, Japan; Department of Gastrointestinal and Pediatric Surgery, Tokyo Medical University, Shinjuku, Tokyo 1600023, Japan; Department of Anatomic Pathology, Tokyo Medical University, Shinjuku, Tokyo 1600023, Japan; Department of Anatomic Pathology, Tokyo Medical University, Shinjuku, Tokyo 1600023, Japan; Department of Gastrointestinal and Pediatric Surgery, Tokyo Medical University, Shinjuku, Tokyo 1600023, Japan; Department of Gastrointestinal and Pediatric Surgery, Tokyo Medical University, Shinjuku, Tokyo 1600023, Japan; Department of Gastrointestinal and Pediatric Surgery, Tokyo Medical University, Shinjuku, Tokyo 1600023, Japan

## Abstract

Colorectal cancer rarely develops pericardial metastasis, and it is an extremely rare case that cardiac tamponade due to the metastasis of colorectal cancer during life. Our case is of a 50-year-old woman who underwent laparoscopic lower anterior resection for the rectal cancer with lung metastasis 4 years ago developed cardiac tamponade due to pericardial metastasis of rectal cancer. We performed pericardiocentesis as a temporary life-saving procedure, but pericardial fluid re-accumulated within a few days. She died 23 days after admission. When a patient with advanced colorectal cancer complains dyspnea, we should consider the pericardial metastasis, and perform the proper treatment as this case.

## INTRODUCTION

Distant metastases of colorectal cancer are often hematogenous, and liver metastasis are the most common affected site [1]. Pericardial metastasis is one of the rarer patterns of hematogenous metastasis of colorectal cancer, but it is very difficult to diagnose before death and even rarer to cause symptomatic cardiac tamponade [2]. We need requiring prompt management within a few hours or days for cardiac tamponade. But there are no established therapy after acute phase. This report describes a rare case of pericardial metastasis of rectal cancer, along with a literature review.

## CASE REPORT

**Figure 1:**
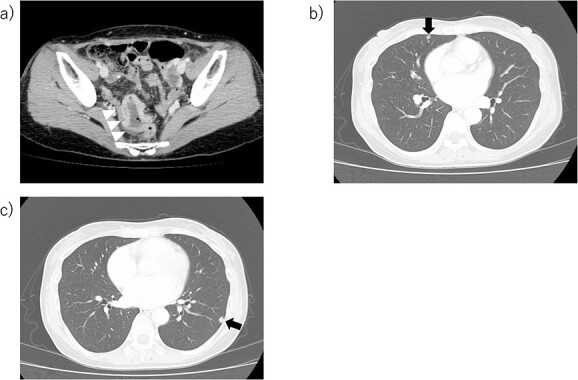
Images at the time of rectal cancer surgery. (A) Circumferential wall thickening, which is contrast-enhanced, is observed in from the sigmoid colon of the anal rectum to the upper rectum (white triangle point). (B, C) Multiple nodules suspected to be rectal cancer metastases in the lung field (black arrow point).

**Figure 2:**
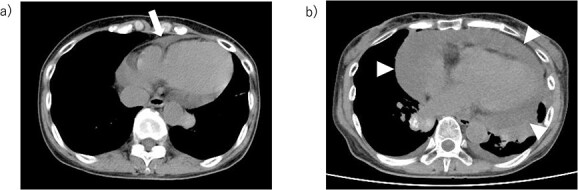
Pericardial thickening. (A) Mild thickening of the pericardium 2 months before cardiac tamponade (white arrow point). (B) Pericardial effusion causing cardiac tamponade (white triangle point).

A 50-year-old woman presented to the hospital complaining of anemia. Her medical history included hyperthyroidism, hypertrophic cardiomyopathy, urolithiasis, and panic disorder. She has no smoking history. She was diagnosed with a circumferential Type 2 tumor of the upper rectum in colonoscopy. Computed tomography (CT) showed thickening of the circumferential wall, with a contrast effect in the upper rectum, a swollen #251 lymph node, and three pulmonary nodules suspected to be metastases (Fig. 1). Laparoscopic lower anterior resection and lymph node dissection were performed to prevent intestinal obstruction due to the tumor. Postoperative chemotherapy was initiated for lung metastases. A CT scan 4 years postoperatively revealed pericardial thickening (Fig. 2). She had no subjective symptoms and only mild pericardial fluid retention, and chemotherapy was continued. Two months after the CT finding, she was brought to the emergency room due to respiratory distress. At admission, she presented with tachycardia and Beck’s triad (hypotension, venous distension and diminished heart sounds). CT scans revealed lung metastases of rectal cancer and accumulation of pleural effusion and pericardial fluid (Fig. 2). Cardiologists confirmed the findings of pericardial fluid retention and a sign of right ventricle collapse on echography.

**Figure 3:**
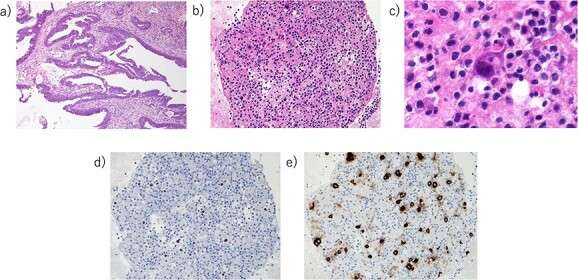
Pathology. (A) Pathology of rectal cancer [hematoxylin eosin (HE) stain]. (B, C) Pericardial fluid (cell block method, HE stain). (D) Pericardial fluid (cell block method, CDX-2 stain). (E) Pericardial fluid (cell block method, CEA(mono) stain).

We diagnosed her cardiac tamponade and performed pericardiocentesis, removing 700 mL of pale yellow drainage. Palpitation and dyspnea improved, and the patient’s breathing calmed down. A few days later, re-accumulation of pericardial fluid was noted. We performed a pathological examination of the collected pericardial fluid (Fig. 3). In cytology, papanicolaou staining revealed atypical epithelial-like cells with a high nuclear-cytoplasmic ratio and hyperchromatic nuclei with irregular shapes. Alcian blue staining revealed a small number of atypical cells with cytoplasmic mucin. In cell block immunohistochemistry, atypical cells were positive for CEA (Mono) and CDX-2, but negative for Calretinin, WT-1 and CD68 (PGM1). These findings were consistent with a diagnosis of pericardial effusion due to pericardial metastasis of rectal cancer. She was started on diuretics, but pericardial fluid quickly accumulated, gradually deteriorating her general condition. She died 23 days after emergency hospitalisation.

## DISCUSSION

Rectal cancer are often diagnosed with the metastasis. The most common mode of metastasis in rectal cancer is hematogenous, with the liver being the most common distant metastatic site, followed by the lungs [1]. This is because, unlike colon cancer, rectal cancer drains from the distal rectal vein through the middle and inferior rectal veins to the internal iliac vein, and thus hematogenous metastasis is likely to occur bypassing the portal hepatic circulation. This may be the reason why the tumor cells directly extended into the pericardial tissue.

Cardiac metastasis of malignant tumors is a relatively common form of metastasis in the terminal stage of cancer. But it is rare to be diagnosed with pericardial metastasis of a tumor during life. This is because many cases of pericardial effusion due to cancer are not accompanied by chest pain and have a poor inflammatory response [2], so they are often not noticed until cardiac tamponade occurs. Symptomatic carcinomatous pericarditis has been reported in male patients with lung cancer and female patients with breast cancer [3], but rarely in patients with colorectal cancer.

Treatment of cardiac tamponade in cancer patients includes systemic chemotherapy, pericardial adhesion therapy, and pericardial incision. Pericardial drainage is often used as a temporary life-saving procedure, but re-accumulation of pericardial fluid occurs in roughly 60% of patients treated with pericardial drainage alone [4]. Thus, many patients continue to require further treatment to prevent pericardial fluid re-accumulation. Pericardial incision may be performed during the first cardiac tamponade in order to prevent pericardial fluid re-accumulation. However, surgery is considered a difficult option due to concerns about surgical tolerance and postoperative quality of life, as many patients are in the terminal stages of the disease. After releasing pericardial fluid retention, systemic administration of antineoplastic drugs may prolong survival. In recent years, pericardial adhesion therapy by anticancer drugs has been performed as a new treatment to address cardiac tamponade. A JCOG study conducted in Japan reported that bleomycin or cisplatin is recommended for lung cancer, and thiotepa or cisplatin is recommended for breast cancer [5]. However, no effective anticancer drugs have been reported for colorectal cancer.

A review of the literature using PubMed (keywords ‘colorectal cancer’ and ‘pericardial metastasis’) identified only five cases [6–10] of cardiac tamponade with an antemortem diagnosis of pericardial metastasis from colorectal cancer, excluding current cases are excluded. In all cases, patients visited the hospital with acute onset respiratory distress. Pericardiocentesis was performed in four of the cases. Surgery was performed with or without pericardiocentesis in three cases; only one was successful, and one died during surgery. Pericardial adhesion therapy was performed in one patient, but the effect was poor. Percutaneous drainage was also performed early in our case, and temporarily relieved the cardiac tamponade. Although early re-accumulation of pericardial fluid was observed after pericardiocentesis in this patient, surgery was not performed because of his general condition and poor prognosis, and the best supportive care policy was adopted.

Advances in medical care have made it possible for patients with distant metastases to survive for a long period of time. When a cancer patient with distant metastasis presents with dyspnea due to pericardial tamponade, it is necessary to not only provide first aid to relieve symptoms but also to submit the collected pericardial fluid for pathological examination to diagnose whether the pericardial effusion is due to pericardial metastasis. And we need to pursue further treatments for long-term survival of colorectal cancer patients with pericardial metastasis.

## CONFLICT OF INTEREST STATEMENT

None declared.

## FUNDING

Not applicable.

## ETHICS APPROVAL AND CONSENT TO PARTICIPATE

Not applicable.

## CONSENT

We explained the clinical trials and disclosure of information to the patient’s family and obtained written consent.

## GUARANTOR

Junichi Mazaki, MD.
